# Elasticity Modification of Biomaterials Used in 3D Printing with an Elastin–Silk-like Recombinant Protein

**DOI:** 10.3390/jfb15060141

**Published:** 2024-05-24

**Authors:** Violetta Cecuda-Adamczewska, Agnieszka Romanik-Chruścielewska, Katarzyna Kosowska, Iwona Sokołowska, Natalia Łukasiewicz, Paulina Korycka, Katarzyna Florys-Jankowska, Agnieszka Zakrzewska, Michał Wszoła, Marta Klak

**Affiliations:** 1 Foundation of Research and Science Development, 01-424 Warsaw, Poland; agaromanik@wp.pl (A.R.-C.); katarzyna.kosowska@fndacjabirn.pl (K.K.); iwona.sokolowska@fundacjabirn.pl (I.S.); natalia.lukasiewicz@fundacjabirn.pl (N.Ł.); korycka.paulina93@gmail.com (P.K.); katarzyna.florys.jankowska@fundacjabirn.pl (K.F.-J.); 2 Polbionica Ltd., 01-424 Warsaw, Poland; agnieszka.zakrzewska@polbionica.com (A.Z.); michal.wszola@polbionica.com (M.W.)

**Keywords:** recombinant proteins, elastin- and silk-like engineered polypeptide, biomaterials, 3D bioprinting

## Abstract

The recombinant structural protein described in this study was designed based on sequences derived from elastin and silk. Silk–elastin hybrid copolymers are characterized by high solubility while maintaining high product flexibility. The phase transition temperature from aqueous solution to hydrogel, as well as other physicochemical and mechanical properties of such particles, can differ significantly depending on the number of sequence repeats. We present a preliminary characterization of the EJ17zipR protein obtained in high yield in a prokaryotic expression system and efficiently purified via a multistep process. Its addition significantly improves biomaterial’s rheological and mechanical properties, especially elasticity. As a result, EJ17zipR appears to be a promising component for bioinks designed to print spatially complex structures that positively influence both shape retention and the internal transport of body fluids. The results of biological studies indicate that the addition of the studied protein creates a favorable microenvironment for cell adhesion, growth, and migration.

## 1. Introduction

One of the rapidly developing trends in the design of new biomaterials for medical applications is the use of recombinant proteins. Such proteins can, for example, act as drug carriers [1,2,3,4], provide protective functions for biologically active molecules, and build or enrich structural proteins used in regenerative medicine [5,6]. To create such chimeric recombinant proteins, structures or functional domains derived from various naturally occurring proteins with known biological functions and defined properties are used. For structural proteins, collagen, elastin, resilin, and silk fibroin are of particular interest [5,7]. Using well-known sequences from elastin and silk fibroin molecules and enriching them with an RGD motif derived from the fibronectin adhesion sequence, we designed a hybrid recombinant structural protein, presented in this paper, for efficient expression in a prokaryotic expression system [7,8].

Among the many proteins and polysaccharides of the extracellular matrix, elastin, one of the main constituents, provides flexibility and structural integrity [9]. Elastins are attractive models for artificial protein-based materials because of their unique physicochemical and mechanical characteristics [10]. Previous studies of ELPs have shown that changes in the sequence space of the hydrophobic and hydrophilic domains and the length [11] of the resulting molecules have a significant effect on the physicochemical, mechanical, and rheological properties [12] of both the proteins and the hydrogels they form and, consequently, on their further biomedical applications [13,14,15]. The most common functional motif of elastin domains used in the design of ELPs (elastin-like proteins) is poly (VPGVG) or a recombinant multiple pentapeptide (VPGXG)_n_, where X can be any amino acid other than proline [16,17]. Materials based on repeated elastin sequences are thermally responsive. They exhibit an inverse phase transition (ITT) at a temperature that is strongly influenced by the identity of the amino acid residue in the pentapeptide repeat [16]. An increase in the X-amino acid residue hydrophobicity lowers the transition temperature, whereas more hydrophilic guest residues increase the temperature. According to research previously presented in the literature, various ELPs have been synthesized to obtain modified molecules exhibiting pH or temperature responsiveness [18]. The substitution of the first glycine by an L-alanine VPAVG modified the properties of the obtained ELPs to a plastic more than to an elastomer. In addition, it demonstrated distinct thermodynamic characteristics in the ITT process [19,20]. The amino acid sequences VPAGG, VPAAG, and VPAFG represent the elastin domains in the final protein described in this work. Basheer A. et al. suggest that these sequences modify the temperature values of LCST and UCST which, in turn, changes the ranges for solubilization and aggregation of the resulting protein molecules [21].

Similarly to elastin, silk is the subject of research into its use in the production of biomaterials with notable strength and elasticity. Spider silk peptide-inspired recombinant proteins have been produced in high yields in various expression systems [22,23,24]. Silk protein-like multiblocks derived from repetitive motifs of *Bombyx mori* and spider dragline silk have the ability to spontaneously aggregate into β-sheet structures present in natural silk fibroins [25]. The (GAGAGS)n polypeptide was also reported to possess a structure that influences the degree of chain polymerization. Domains containing repetitions of the hexapeptide GAGAGS from the silk fibroin heavy chain [26,27] have been added.

The design of the recombinant hybrid protein was enriched for functional domains. The RGD motif (AVTGRGDSPASS) was borrowed from the human fibronectin integrin-dependent cell adhesion sequence [28] to improve cell–recombinamer interaction. According to the literature, the addition of fibronectin-derived RGD domains to biomaterials promotes cell adhesion (e.g., in endothelial cells [29], smooth muscle cells [30], and fibroblasts [31,32]). Therefore, recombinant hybrid protein sequences enriched with an RGD motif may favorably influence the growth process of cells [33]. The sequence of the ZIP domain, which encodes the stabilizing supramolecular structure of the leucine zipper, was also used in the designed recombinant protein. Enrichment of the recombinant structural proteins with the basic leucine zipper (bZIP) dimerization domain of HLF (*hepatic leukemia factor*) [34] was expected to provide structural stability during printing due to its ability to form amphiphilic α-helical structures based on hydrophobic interactions [28]. Through the use of the domain combinations described above, the developed protein should be applicable not only for the remodeling of polymeric biomaterials but also for the mediation of cell colonization, migration, and proliferation and for the stimulation of new tissue growth.

**Figure 1:**
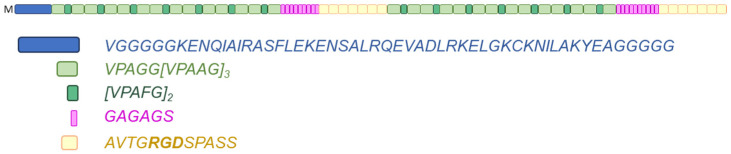
Scheme of the modular structure of the EJ17zipR protein (top). HLF leucine zipper (dark blue), elastin repetitive sequences (green ones), silk fibroin sequences (pink), and fibronectin RGD motifs (yellow).

The EJ17zipR hybrid protein described in this work is sequentially constructed from one ZIP domain, fifty-six elastin domains, nine silk fibroin domains, seven RGD domains, fifty-six elastin domains, ten silk fibroin domains, and seven RGD domains. Interestingly, in the amino acid sequence of the engineered EJ17zipR protein, glycine represents up to 26% and proline almost 15% of the total amino acid residues. The molecular mass of the full-length protein is approximately 86 kDa. A block diagram of the recombinant EJ17zipR protein is shown in Figure 1. The complete amino acid sequence of the protein presented in this paper can be found in the Supplementary Materials section (Figure S2). The synthetic recombinant protein EJ17zipR can be successfully used in many applications, including as an addition to bioinks used in 3D bioprinting technology for tissue models, thus improving their functional properties such as viscosity, printability, and mechanical parameters, as well as biocompatibility and biodegradability.

## 2. Materials and Methods

### 2.1. DNA Constructs

The nucleotide sequence of the hybrid protein gene was designed using SnapGene software and optimized for the host cell, an E. coli strain. The DNA sequence was ordered from GeneScript Biotech (Rijswijk, The Netherlands) and cloned directly into the pET11a expression vector. The plasmid contains a T7 phage-derived promoter, a lac operon, and an ampicillin resistance gene sequence. The resulting expression vector allows for efficient and stable expression of recombinant proteins in E. coli cells by induction with lactose or its analog (e.g., IPTG: isopropyl-β-d-1-thiogalactopyranoside). A schematic map of the expression vector is provided in the Supplementary Materials section (Figure S1).

### 2.2. Polypeptide Expression

Plasmid E. coli cells of the BLR(DE3) strain (Novagen, Merck, Darmstadt, Germany) were chemically transformed with EJ17zipR-pET11a and cultured in flasks in 500 mL of LB medium supplemented with antibiotic (50 mg/L ampicillin) at a temperature of 30 °C. This was placed in a microbiological shaker and orbital-agitated at a speed of about 150 rpm until an optical density of OD600 0.7–0.9 was reached. Expression of the recombinant protein was then induced by adding IPTG (Sigma-Aldrich, St. Louis, MO, USA) to a final concentration of 0.2 mM. The culture was continued for 6 h at 37 °C and shaken at 200 rpm until the optical density of the culture, measured as OD600, reached a value of approximately 3.

### 2.3. Protein Purification

The bacterial biomass was separated from the culture medium by centrifugation (8000 rpm, 4 °C, 10 min.). The bacterial cells pellet was resuspended in lysis buffer consisting of 0.5 M NaCl, 50 mM Tris-HCl pH 7.5, 10 mM EDTA pH 8.0, and 5 mM ẞ-mercaptoethanol, with the protease inhibitors mix (0.35 mg/mL lysozyme, 0.7 mM PMSF, and 10 mM benzamidine). The suspension was sonicated on ice at an amplitude of 33% for 10 cycles of 10 s each (SONOPULS ultrasonic homogenizer, sonotrode, 13 mm, Bandelin, Berlin, Germany) to disintegrate cell walls and release proteins from the cell structures. An amount of 0.4% (*w*/*v*) polyethyleneimine (PEI, Sigma-Aldrich, St. Louis, MO, USA) was added to the suspension to precipitate the host DNA in this step. Separation of the insoluble protein fractions, nonintegrated bacterial cells, and precipitated DNA from the supernatant containing the recombinant hybrid protein was enabled by centrifugation. The denatured proteins were centrifuged again after incubation of the supernatant with the EJ17zipR protein at 90 °C for 20 min. At this stage, a protease inhibitor mixture (cOmplete, EDTA-free Protease Inhibitor Cocktail, Roche Diagnostics, Mannheim, Germany) was added to the recombinant protein solution, in the amount of one tablet per 50 mL, to protect the released proteins. The recombinant EJ17zipR protein was precipitated from protein mixture by desalting with 10% ammonium sulfate, at room temperature, next separated from the solution via centrifugation, and the resulting protein pool was dissolved in 20 mM Tris buffer with 10 mM EDTA pH 8.0. Ammonium sulfate contents was removed via overnight dialysis of the recombinant protein suspension into 20 mM Tris buffer with 10 mM EDTA pH 8.0 at 4 °C.

The protein was purified on an FPLC system using a Macro-Prep High Q Media resin (Bio-Rad Laboratories, Hercules, CA, USA). The protein solution obtained via solubilization of the salted protein was applied to the column equilibrated with 20 mM Tris calibration buffer pH 8.0. Unbound proteins were eluted with the calibration buffer. Proteins bound to the resin were eluted with elution buffer consisting of 20 mM Tris pH 8.0 with the addition of 1 M NaCl. A flow rate of 1–2 mL/min was used during the separation, and fractions were collected according to an absorbance reading value of at least 0.05. The eluted protein concentration was estimated using both the Bradford and BCA methods (Pierce BCA Protein Assay Kit, Thermo Fisher Scientific Inc., Rockford, IL, USA). It should be noted that the recombinant protein EJ17zipR did not bind to the applied resin and was eluted from the column with calibration buffer. In the next purification step the resulting protein was purified from endotoxins using Pierce High-Capacity Endotoxin Removal Resin columns (Thermo Fisher Scientific Inc., Rockford, IL, USA) according to the manufacturer’s instructions. The isolate of final protein was dialyzed to ddH_2_0 for 24 h at 4 °C. The resulting protein was lyophilized and may be long-term stored at 4 °C.

The purification process of the recombinant EJ17zipR structural protein was controlled at each stage by means of SDS-PAGE electrophoresis under denaturing conditions. The expression yield was approximately 30–60 mg of recombinant EJ17zipR from 1 L of flask culture.

### 2.4. Amino Acid Sequence via Mass Spectrometry Analysis and Purity Confirmation (HPLC Analysis)

Mass spectrometry experiments were performed at the Mass Spectrometry Laboratory at the Institute of Biochemistry and Biophysics PAS. The conditions for analysis are described in detail in the Supplementary Materials section. To confirm the amino acid sequence, the sample was resuspended in 100 μL of 0.1% TFA in H_2_O. Cysteines were then reduced by incubation with 20 mM tris(2-carboxyethyl)phosphine (TCEP) at 37 °C for 1 h, followed by incubation with 50 mM methyl methanethiosulphonate (MMTS) at room temperature for 10 min. Digestion was carried out on immobilized pepsin (Thermo Scientific, Rockford, IL, USA) at RT for 3 h. After digestion, peptides were acidified with 0.1% formic acid.

Analysis was performed using an LC-MS system comprising an Evosep One (Evosep Biosystems, Odense, Denmark) coupled to an Orbitrap Exploris 480 mass spectrometer (Thermo Fisher Scientific, Bremen, Germany) via a Flex nanoESI ion source (Thermo Fisher Scientific, Bremen, Germany). Samples were loaded onto disposable Evotips C18 trap columns (Evosep Biosystems, Odense, Denmark) in accordance with the manufacturer’s protocol with minor modifications. Chromatography was performed at a flow rate of 250 nL/min using the 88 min (15 samples per day) preformed gradient on the EV1106 analytical column (Dr Maisch C18 AQ, 1.9 μm beads, 150 μm ID, 15 cm long, Evosep Biosystems, Odense, Denmark). Raw data were preprocessed with the Mascot Distiller software (v. 2.4.2.0; Matrix Science Ltd., London, UK); then, the obtained peptide masses and fragmentation spectra were matched to the E. coli database (4403 sequences; 1,354,446 residues), cRAP (115 sequences; 38,188 residues), and User database. When the modifying sequences were added, the Mascot search engine (Mascot Daemon v. 2.4.0, Mascot Server v. 2.4.1, and Matrix Science) was used. Protein identification was performed using the Mascot search engine and the probability-based algorithm. The Decoy Mascot functionality was used for maintaining FDR below 1% for peptide identifications.

Purity analysis of the tested protein was carried out via UHPLC on a Waters AQUITY instrument with a PDA eλ detector. The chromatogram was monitored at 3 wavelengths (λ) (200, 210, and 220 nm) to optimized the analysis conditions, with the best results obtained at 200 nm. Finally a Waters’ SunFire C8 3.5 μm, 2.1 × 100 mm column was chosen for the analysis. The products of separation were eluted with a binary phase layout of 0.1% TFA (trifluoroacetic acid) in water and 0.1% TFA in acetonitrile, at a flow rate of 0.5 mL/min, and with a column temperature of 30 °C.

### 2.5. Physicochemical Tests of Biomaterials with Addition of EJ17zipR—Assessment of the Usefulness of Biomaterials Enriched with the EJ17zipR Protein

#### 2.5.1. Preparation of Material Based on GelMa Hydrogel and dECM Bioink Enriched with EJ17zipR Recombinant Protein

The purified EJ17zipR protein after lyophilization was used to create a bioink with high utility in the technology of bioprinting three-dimensional constructs. In the first approach, the bioinks were prepared as a composition of 2 components of the recombinant EJ17zipR protein dissolved in PBS and 10% (*w*/*v*) methacrylated gelatin (TINTBIONIC GELMA 80; Polbionica Ltd., Warsaw, Poland) in 1× PBS with 1.85 mg/mL LAP photoinitiator (phenyl-2,4, Lithium 6-trimethylbenzoylphosphinate (Polbionica Ltd., Poland). Three concentrations of recombinant protein in the biomaterial were tested 0.1, 0.5, and 1.0 mg/mL and compared to the base material containing 10% GelMa and LAP. In the second part of the experiment, an enriched version of the bioink containing the dECM extracellular matrix (Printiss^®^ dECM-PAN; Polbionica Ltd., Warsaw, Poland), obtained from the pancreas with the addition of recombinant EJ17zipR protein, was used. In addition to dECM-based bioink (81.27 mg dECM/mL bioink), the tested material contains methacrylated gelatin (TINTBIONIC GELMA 80; Polbionica Ltd., Poland) (37.15 mg/mL), methacrylated hyaluronic acid (TINTBIONIC HAMA; Polbionica Ltd., Poland) (5.57 mg/mL), and LAP (2.32 mg/mL). The recombinant EJ17zipR protein was used in the experiment at two concentrations: 0.1 and 1.0 mg/mL. Recombinant EJ17zipR protein was dissolved in methacrylated hyaluronic acid. The reference sample was the same material without the addition of recombinant protein.

#### 2.5.2. Rheology

The rheological properties of the developed material were tested using an Anton Paar MCR 72 rheometer. Three rheological parameters were determined. The measurement of the complex modulus, depending on the temperature change, was performed under conditions of 5% deformation, 1 Hz frequency, and in the temperature range of 10–35 °C. The measurement of the complex modulus, depending on the change in the set strain from 1 to 100%, was performed at a temperature of 20 °C and a frequency of 1 Hz. The rotational measurement of dynamic viscosity was performed at a constant temperature of 25 °C and a constant shear rate of 100 s^−1^. All tests performed for the tested materials were performed using a PP25 spindle, and the gap was set to 1 mm. Based on the results, the sol–gel phase transition point, the dependence of the storage modulus and loss modulus on shear stress, and the average viscosity at a given temperature at a constant shear rate were identified.

#### 2.5.3. Printability of Materials Enriched with the EJ17zipR Recombinant Protein

The printability of the developed biomaterials was tested. For this purpose, a specially developed procedure of a three-stage assessment system was used: a fusion test of fibers printed in the form of a template, a collapse test of a fiber printed on a three-dimensional platform, and an assessment of fiber continuity during continuous bioink printing in a volume of 3 mL. In order to select appropriate conditions for printing materials, a number of extrusion tests were performed under various temperature and pressure conditions. Based on the data obtained (observations of the continuity of the obtained fiber), optimal printing conditions for a given material were selected, including pressure, temperature, and printing speed. In order to carry out the fiber fusion test, a model was developed according to which two layers were printed one after the other using the tested material, without the use of cross-linking, with an external lamp between them. Prints were made using a BIO X™ printer (Cellink, Gothenburg, Sweden). The prepared print follows a 0°–90° pattern, reproducing the 2D effect and increasing the fibre distance (FD). The fibre spacing ranged from 1 to 5 mm with 1 mm increments being used. The printing speed of 20 mm/s, needle diameter of 0.609 mm, and printing distance of 0.8 mm were used in the test. The print cross-linking was made with an external UV–Vis lamp of 365 nm for 15 s with a power of 13 W/cm^2^. Based on the results, two parameters described by equations were determined, i.e., the percentage of diffusion rate (material spreading rate) (*D_fr_*) and printability (*P_r_*):

$$D_{fr}=\frac{A_{t}-A_{a}}{A_{t}}\cdot 100\%$$

$$P_{r}=\frac{L_{a}^{2}}{16\cdot A_{a}}$$

where *A_t_* is the theoretical pore surface, *A_a_* is the actual pore surface, and *L_a_* is the actual pore circumference.

Deflection of the suspended fiber was analyzed to determine the material’s collapse affinity. In order to carry out the experiment, a special platform was designed and 3D-printed. This platform consisted of seven pillars spaced apart from each other at known distances of 1, 2, 3, 4, 5, and 6 mm. The five posts placed inside the structure had dimensions of 2 × 10 × 6 mm^3^. The two edge posts had dimensions of 5 × 10 × 6 mm^3^. Using BIO X™ printer (Cellink, Gothenburg, Sweden) the technique of bioprinting, a single fibre of the tested material was deposited on the platform. During printing, temperature and pressure conditions were changed depending on the tested material. The print was made at a speed of 20 mm/s using a 0.609 mm nozzle. The collapse area factor (*C_f_*), which is the percentage of the actual area after the suspended fiber has been deflected from the theoretical area, was calculated using the following equation:

$$C_{f}=\frac{A_{a}^{c}}{A_{t}^{c}}\cdot 100\%$$

where $A_{a}^{c}$ is the actual area under the curve, and $A_{t}^{c}$ is the theoretical area under the curve.

Fiber continuity when printing 2–3 mL of the tested bioink was assessed using the 0/1 system, assuming that when the fiber breaks, the value is 0, and when it is pulled, the value is 1.

#### 2.5.4. Testing of Mechanical Parameters via Static Compression Test

The mechanical compressive strength of the samples was tested using a static compression test. In order to carry out the experiment, cylindrical samples with dimensions were used: d (diameter) of 10 mm and h (height) of 5 mm. These samples were printed on a BIO X™ printer (Cellink, Gothenburg, Sweden) (100% filling, cross-linking with an external UV–Vis lamp after each layer). All samples were initially loaded with a force of 0 to 0.05 N. The samples were compressed at a constant speed of 10 mm/min at room temperature until 80% deformation was obtained. Based on the results, the mechanical strength of the samples was calculated as the maximum stress, the Young’s modulus was calculated as the slope coefficient of the simple relationship between stress and strain of the sample in the strain range of 0.1–0.5, and the conventional yield limit RE0.01 was calculated as the stress causing permanent deformation of the sample at 0.01% of the height.

#### 2.5.5. Water Absorption Capacity of the Tested Biomaterials

The biomaterial was poured onto weighed dishes in three repetitions and cross-linked with light with a wavelength of 365 nm, a power of 13 mW/cm^2^, and a time of 20 s. The sample was weighed again to obtain the mass of the material after cross-linking (WM). Deionized water was added to the dishes, and the covered with parafilm samples were left at room temperature for 24 h. Next the water was removed, and the dishes were dried and weighed again. Then, water was added again, the samples were parafilmed, and left for another 24 h at room temperature. Weighing and replacing deionized water was repeated after 48 and 72 h. The degree of water absorption (described as water content per mg of cross-linked material) was calculated using the following equation:

$$degree\;of\;water\;absorption\;[\frac{{mg}_{H2O}}{{mg}_{biomaterial}}]=\frac{W_{N}-W_{M}}{W_{M}}$$

where *W_N_* is the mass of the biomaterial after soaking at a given time point, and *W_M_* is the mass of the biomaterial after pouring and gelling.

### 2.6. Biological Characterization: Adhesion, Proliferation, and Viability/Cytotoxicity Tests

#### 2.6.1. Cell Culture

The monolayer cell line L929, mouse fibroblasts (ATCC nr kat. CCL-1), was cultured in DMEM (ATCC) supplemented with 10% FBS, 4 mM l-glutamin, 4.5 g/L glucose, 1 mM sodium pyruvate, 1500 mg/L sodium bicarbonate, and penicillin/streptomycin solution under standard conditions (5% CO_2_ and 37 °C).

Cell banks of six to ten passages each were established after the L929 cell line was purchased. Prior to the experiment, the cell banks were thawed and passaged three to four times until a sufficient number of cells were obtained. During the cell culturing, the timing of cell division was verified prior to the experiment to ensure that cell division was occurring at a similar rate during the test.

#### 2.6.2. Cell Adhesion and Proliferation Testing

The AlamarBlue™ Cell Viability Reagent (Invitrogen, Eugene, OR, USA) was used in both tests. The reagent indicates the amount of metabolically active live cells. The assay is based on the conversion of blue resazurin under reducing conditions to resorufin, a dye that emits strong red fluorescence. This fluorescence is measured at an excitation wavelength of 530 nm and an emission wavelength of 590 nm.

In both experiments, 96-well microplates with an unmodified surface designed for suspension-type cell culture (Greiner Bio-one, Frickenhausen, Germany) were used. Fibronectin applied to the well surface coating of the wells at a density of 1 μg/cm^3^ was used as a positive control. A few wells on each plate were not coated with any protein and were used as a negative control. Selected wells were coated with an aqueous solution of EJ17zipR protein at 1 and 5 μg/cm^3^ and dried overnight under sterile conditions. For the adhesion assay, L929 cells were seeded onto the plates at a density of 1 × 10^4^/well. Cells were incubated for 2, 4, and 24 h under standard conditions in dedicated culture medium. After this time, the culture wells were washed with sterile phosphate-buffered saline (PBS buffer) to remove non-adherent cells. In contrast, for the proliferation experiment, L929 cells were seeded at 5 × 10^3^/well. Cells were cultured for longer periods: 2, 24, and 48 h. Culture wells were then washed with PBS buffer to remove dead cells. After the specified time, AlamarBlue reagent was added to the wells and incubated for 3 h. Then, fluorescence measurement was carried out.

#### 2.6.3. EJ17zipR Cytotoxicity Testing

The cytotoxicity study was performed using two methods: the classic MTT assay and calcein AM staining (LIVE/DEAD Viability/Cytotoxicity Kit for mammalian cells, Invitrogen). The cytotoxic effect of the EJ17zipR protein on cells of the L929 line was tested in the MTT assay according to ISO 10993-5:2009(E) Biological evaluation of medical devices. Part 5: In vitro cytotoxicity test. Depending on the planned exposure time, cells were seeded into 96-well plates at densities of 1 × 10^4^/well, 5 × 10^3^/well, and 2.5 × 10^3^/well. The cells were cultured for 24 h under standard conditions in supplemented DMEM to allow fibroblasts to adhere to the bottom of the wells. The assay was performed, using the direct method, by adding purified reconstituted protein solution directly to the culture medium at concentrations of 1, 0.5, and 0.1 mg/mL. A plate containing cell culture at a density of 1 × 10^4^/well was incubated for 24 h. A culture at an initial density of 5 × 10^3^/well was exposed to EJ17zipR protein in medium for 48 h, while cells at an initial density of 2.5 × 10^3^/well were tested with EJ17zipR solutions for 72 h. After the indicated time, the cells were incubated with the MTT solution for 2 h. All liquid above the cells was removed and the resulting formazan salt crystals were dissolved with DMSO. The amount of the resulting colored product, proportional to the number of living cells, was determined by measurement of the absorbance at 570 and 650 nm wavelengths. According to the standard, the expected result is a viability of cells exposed to the cytotoxic agent of not less than 70% of all untreated cells in the negative control.

In the second method, the purified EJ17zipR protein obtained after lyophilization was used to coat 96-well microplates dedicated for the suspension culture of eukaryotic cells (unmodified surface). An aqueous solution of EJ17zipR protein at a final density of 1 μg/cm^3^ was applied to the plates and dried. Fibronectin, also at 1 μg/cm^3^, was used as an assay positive control. L929 cells were seeded at a density of 2 × 10^4^/well, 1 × 10^4^/well, and 0.5 × 10^4^/well in the plates prepared the day before the test. Cells were incubated for 24, 48, and 72 h, respectively, in dedicated culture medium. Finally, cells were stained with calcein AM (1 μM) for 30 min, and fluorescence was measured at wavelengths of 485 nm (excitation) and 530 nm (emission). Nonfluorescent acetylmethoxycalcein (AM calcein) freely permeates cell membranes and is enzymatically converted by intracellular esterases to intensely fluorescent green calcein.

When the cytotoxicity of the EJ17zipR-enriched biomaterial was tested with MTT indirect testing, extracts of fragmented biomaterials were prepared by depositing them on inserts for 24 h in DMEM culture medium. Next, the extracts and positive control (CP, 0.1% Triton/DMEM) were added to the cells seeded into 96-well plates at a density of 5 × 10^3^/well, 2.5 × 10^3^/well, and 1.25 × 10^3^/well and incubated at 37 °C and 5% CO_2_ in DMEM medium for 24 h, 48 h, and 72 h, respectively. To perform a cytotoxicity test the medium/extracts were removed, and 1 mg/mL MTT was added.

### 2.7. Statistical Analysis

The significant difference from the respective controls for each experimental test condition was assessed with a one-way analysis of variance (ANOVA) and the Dunnett test. The difference was significant if the *p*-value was less than 0.05. Statistical analysis was performed using GraphPad Prism V5.01 software (GraphPad Software Inc., La Jolla, CA, USA). Tested samples were analyzed at a given time point compared to a control sample in all experiments requiring statistical analysis.

## 3. Results and Discussion

The molecular mass of the full-length EJ17zipR protein is approximately 86 kDa, while the protein oligomerizes into macromolecules of over 1 mDa, as verified by polyacrylamide gel electrophoresis under native conditions. The obtained recombinant EJ17zipR protein in a concentration less than 10 mg/mL did not form hydrogels in the temperature range from 4 to 99 °C.

A complex multistep purification process resulted in protein preparations with a purity of greater than 98%, as confirmed via UHPLC-MS. We hypothesize that this high level of purification is due to the effective use of the thermostability of ELPs in the recombinant EJ17zipR protein separation process. In a single step, a mixture of proteins released from the bacteria cells was heated to 90 °C, resulting in sedimentation of denatured contaminations, while the soluble EJ17zipR remained in solution. Various strategies to purify ELPs using ITT have been published, such as alternating hot centrifugation above UCST followed by cold centrifugation below LCST, where gel transitions allow protein separation without chromatography [35,36]. However, such a procedure did not work in the case of EJ17zipR, as we observed no gel formation by this protein in the temperature range of 4–99 °C. Only zol formation (opalescence) was observed at about 30 °C. This effect was reversed by lowering the temperature. Of course, the value of the phase transition temperature depends on many factors, including the molecular weight of the peptide [37,38]. It has been observed that the longer the ELP molecule is, the lower the T. This is due to more stable hydrophobic interactions, which disrupt the peptide backbone hydrogen bonding with water [38]. The work of Desai et al. clearly showed the dependence of the transition temperature on the mass of the ELP molecule, where an increase in the peptide mass between 21 and 42 kDa resulted in a decrease in the transition temperature from 40 to 31 °C [39]. The EJ17zipR protein has a molecular mass of 86 kDa, and 67% of the amino acid residues in the molecule are elastin-inspired sequences. We can therefore assume that a temperature of around 30 °C is a probable transition temperature for our protein; nevertheless, it was not possible to successfully centrifuge the desired protein under these conditions. The multistep purification scheme used for the EJ17zip protein was due in part to the decision not to add one of tags commonly used in the pharmaceutical industry [36] (e.g., His_6_-tag, poly arginine tag, GST, or FLAG) to the designed molecule. From the outset of the project, it was assumed that tag removal would require additional enzymatic digestion-like steps that could significantly increase the cost of purification and introduce an additional protein in the form of an enzyme to be removed from the final product. Despite the absence of the tag, it was possible to optimize a purification scheme that guaranteed high protein purity in a process monitored at each step in SDS-PAGE electrophoresis.

After digestion of the samples with pepsin and analysis via mass spectrometry, the amino acid sequence of the resulting protein was confirmed by peptide mapping. For the EJ17zipR test sample, a result close to 100% coverage was obtained compared to the designed sequence. Due to the strong tendency of EJ17zipR to oligomerize, the correct confirmation method for the molecular weight of the test protein was not found. The mass of the oligomer was too large to be measured using mass spectrometry. Unfortunately, none of the tested methods (0.1% TCA, reduction by 1 h with 20 mM TCEP or n-Dodecyl-β-d-maltoside) of dissolving the EJ17zipR-R protein into a monomer produced good results. As mentioned in the introduction, when elastin sequence pentamer variants containing one of the hydrophobic amino acids as the fourth residues are used in ELP molecules, the resulting peptides or proteins show a strong tendency to oligomerize [9,21]. In the protein described, the fourth hydrophobic amino acid in each variant was glycine, alanine, or phenylalanine. Therefore, the formation of very large oligomers was not a surprising result. This effect was confirmed by gel electrophoresis under non-denaturing conditions, where EJ17zipR protein aggregates remained in the upper stacking part of the gel.

**Figure 2:**
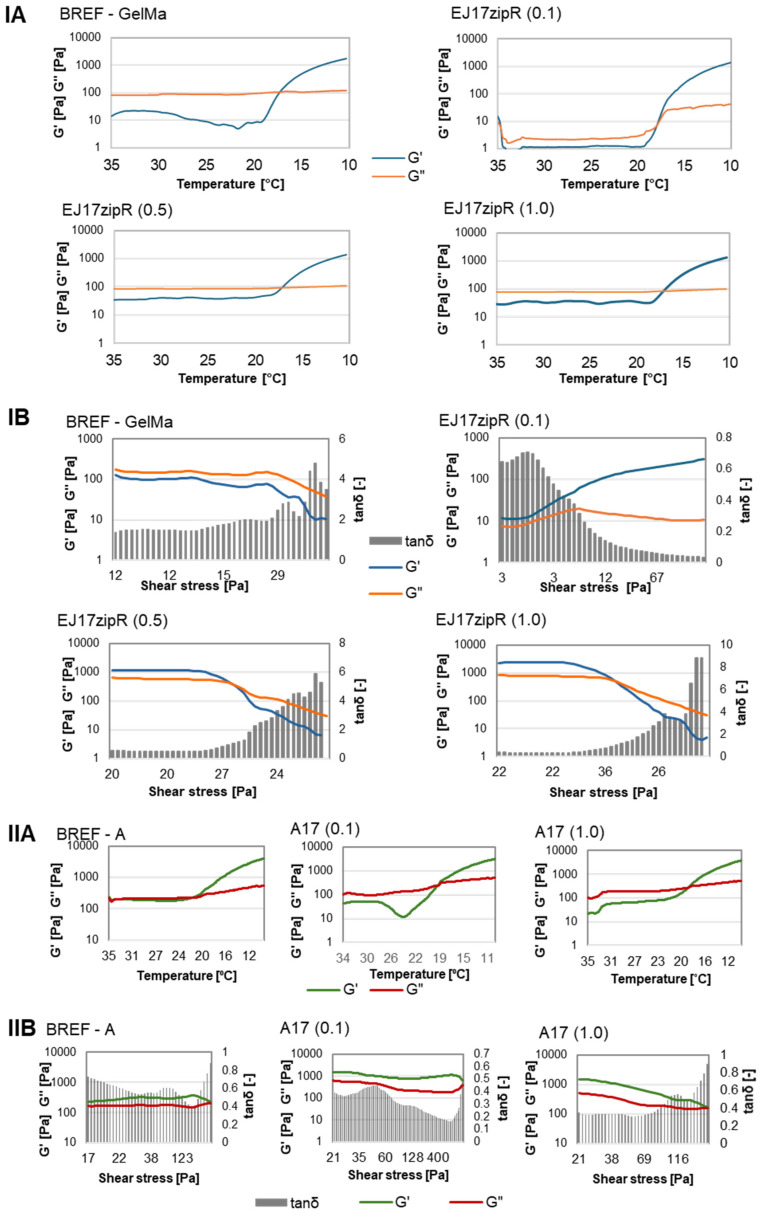
The rheological property study of the tested materials based on GelMa or dECM containing the EJ17zipR recombinant protein in comparison to reference bioink (10% GelMa is designated as BREF, dECM-containing bioink is designated as BREF—A). (**IA** for GelMa and **IIA** for dECM-bionk respectivelly) Sol–gel phase transition point, (**IB** for GelMa and **IIB** for dECM-containing bioink) complex module.

Hybrid silk–elastin copolymers are being tested to improve the solubility of the product while maintaining a high degree of flexibility [40]. At selected concentrations, such biomaterials are able to transition from aqueous solution to hydrogel due to the formation of a β-sheet structure in the silk domains. The transition temperature and other physicochemical and mechanical properties of such molecules can vary significantly depending on the number of sequence repeats in both elastin and silk [41,42]. The aim of this work was to obtain a protein that would effectively help modify the properties of the basic components of bioinks used in 3D printing. The optimal bioink should have biological, rheological, and mechanical properties as similar as possible to those of the target tissue. The rheological properties of the bioink are a critical property, as they have an impact on the proliferation, differentiation, and apoptosis of the cells [43]. As a result, the viscosity and thinning properties need to be adjusted to support the 3D printing extrusion process and ensure the structural stability of the printed constructs without reducing cell viability [44]. In addition, the gelation time has to be regulated in order to create self-supporting constructs, while the matrix itself has to provide a biological environment that promotes cell viability and the exchange of nutrients and metabolic intermediates. High-water-content, porous 3D hydrogel networks offer optimal properties to mimic soft tissue constructs for tissue engineering and regeneration [45]. The physical, chemical, and biological properties of these hydrogels can be tailored for design purposes [46]. The obtained results indicate that the addition of recombinant protein to methacrylated gelatin does not affect the temperature of the sol–gel phase transition point, which is 17 °C for both the reference sample and the tested material. The addition of the recombinant protein EJ17zipR, regardless of the concentration, modified the dependence of the complex modulus on the shear stress. For the reference sample, in the entire strain range tested, the loss modulus was higher than the storage modulus. However, for biomaterials containing the EJ17zipR recombinant protein, initially, the storage modulus was higher than the loss modulus, increasing the set deformation resulted in a change in the relationship, and the viscous properties (G″) prevailed over the elastic properties (G′). When it comes to the possibility of using a material for printing 3D models, a more important parameter, apart from viscosity, is the storage modulus rather than the loss modulus. It was observed that the addition of recombinant protein to 10% GelMa resulted in a decrease in dynamic viscosity compared to the reference sample (Figure 2). The viscosity of the hydrogel containing the recombinant protein EJ17zipR(0.1) is 13.234 Pa·s; for EJ17zipR(0.5), it is 15.057 Pa·s; for EJ17zipR(1.0), it is 17.986 Pa·s; and for the BREF GelMa reference sample, it is 24.11 Pa·s. Increasing the concentration of the EJ17zipR protein increases the viscosity of the material, but the reference sample exhibits the highest viscosity.

All the viscosity values obtained for materials containing the recombinant EJ17zipR protein are acceptable for the potential use of the material in 3D printing, while the addition of the protein is expected to have a further beneficial effect on cells. The results obtained in the experiment with bioink A also indicate the lack of influence of the addition of the recombinant EJ17zipR protein on the dynamic viscosity and the sol–gel phase transition point compared to the reference bioink A. The viscosity of the dECM-based material containing the A17(0.1) protein is 0.301 Pa·s and A17(1.0)–0.521 Pa·s, while, for the reference sample without the protein, the viscosity is 0.464 Pa·s. The addition of the recombinant protein resulted in an increase in the values of the complex module components compared to the reference bioink A. Such rheological properties are both used for extrusion bioprinting and do not have a negative impact on cell proliferation and differentiation. Measurement results are shown in Figure 2.

**Figure 3:**
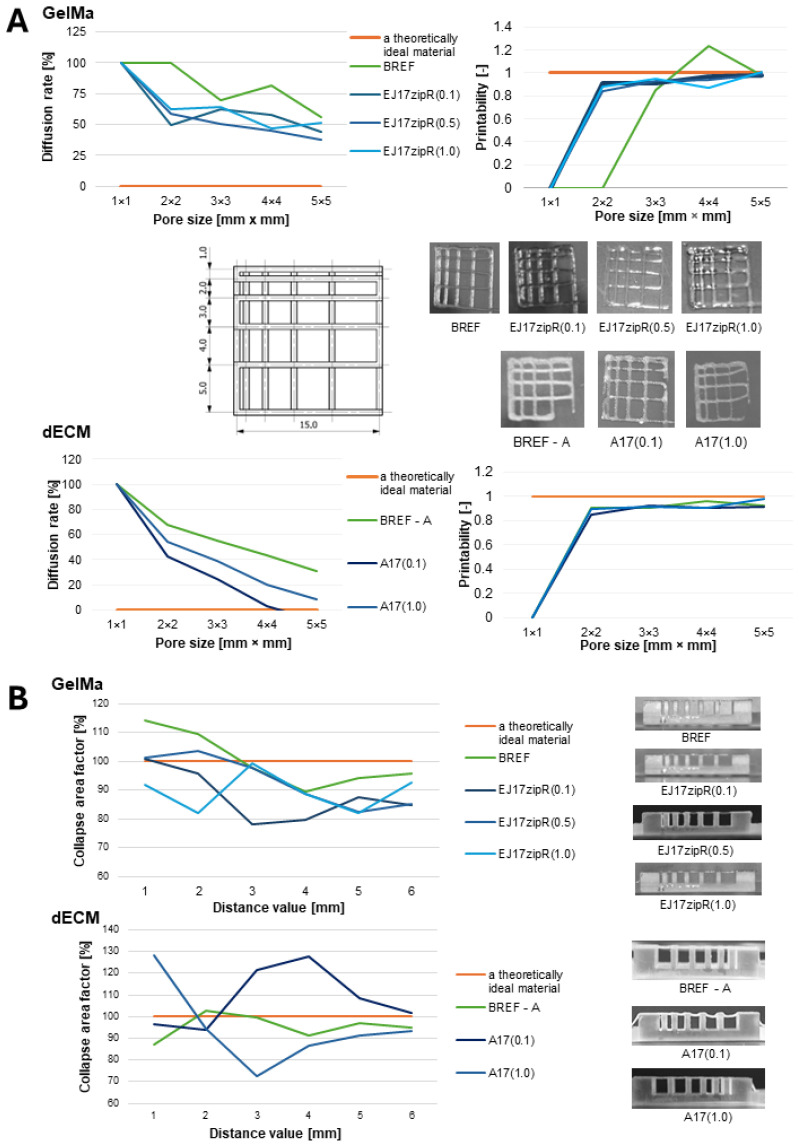
The printability of biomaterials based on GelMa or dECM containing the recombinant ZJ17zipR protein in comparison to 10% GelMa (BREF) or dECM-containing bioink (BREF—A); (**A**) diffusion rate and printability, (**B**) fiber collapse coefficient.

The results of the fiber collapse test as a measure of the fiber’s stability are shown in Figure 3. Both materials based on 10% GelMa and dECM bioink containing EJ17zipR recombinant protein were characterized by fiber continuity and stability. The collapse coefficient was over 80% for GelMa and 70% for the dECM bioink. A fiber fusion test was also performed as a measure of the material’s printability and resolution. Based on the results, it was found that the diffusion rate decreases and the printability increases as the size of the pores on the template increases. Again, both 10% GelMa with the addition of the recombinant EJ17zipR protein and the dECM bioink enriched with this protein are characterized by a high printing resolution, clearly better than the material without the additive. A material with such features as an addition to bioink could be used when it is necessary to print relatively small objects with a large amount of detail, due to the high printing resolution and the lack of uncontrolled spread of the material. One of the possible applications being considered is the use of controlled drug dosing systems or tissue models with a vascular system in bioprinting. As a result of testing the continuity and smoothness of the fiber, it was found that the tested materials are printable within the range of the following printing parameters: a temperature of 20–22 °C and a pressure of 35–50 kPa for 10% GelMa and a temperature of 23–25 °C and a pressure of 40 kPa for bioink A with dECM. It is worth mentioning that these printing parameters, such as temperature and pressure, are important for printing tissue models. Our materials can be printed under relatively mild conditions which are considered to be hospitable to cells [47]. Similar research was conducted by other authors [48,49,50,51,52]. The addition of the EJ17zipR protein allows us to achieve better printability parameters, which is closely related to the rheological properties of the material, as well as the molecular structure of the composite. The use of structural proteins in bioprinting technology supports the structural organization and stabilization of the printed fibers due to the self-organization and cross-linking of the component proteins, which also affects the mechanics and function of the printed tissue. The literature has investigated the use of both synthetic and natural hydrogels in 3D bioprinting [53]. Natural hydrogels are more effective in reproducing the properties of the native tissue environment. Their main advantages include achieving bonding for cell–matrix interactions, biodegradability, and lack of toxicity. In addition, natural polymers, e.g., structural proteins, are characterized by a high molecular weight, which leads to the formation of gels with relatively high viscosity at lower protein concentrations and reduces cell hindrance compared to synthetic biodegradable materials [54].

**Figure 4:**
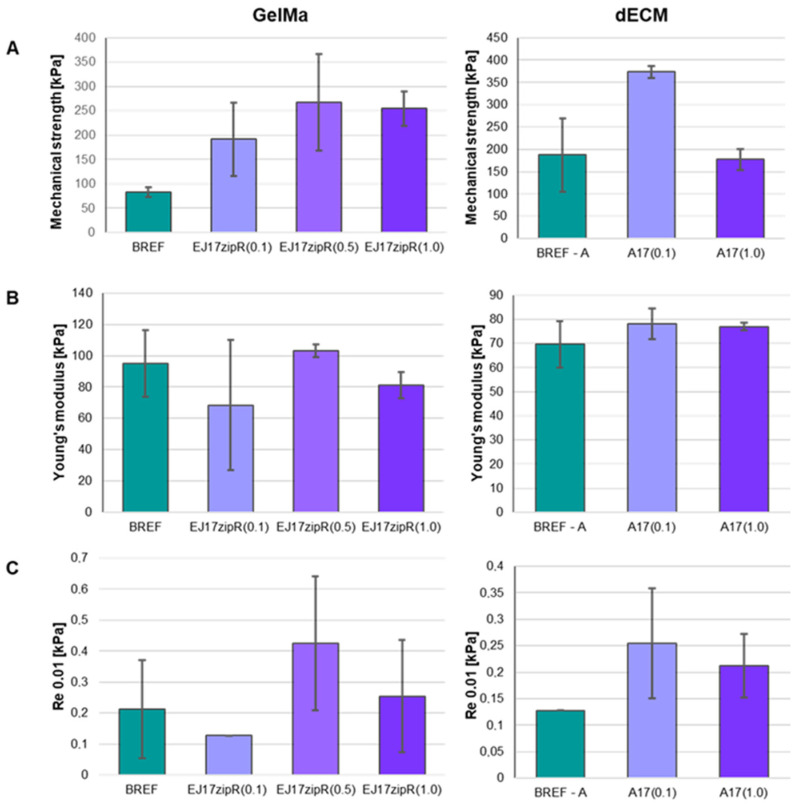
Mechanical parameters of bioinks based on 10% GelMa containing ZJ17zipR protein and dECM and EJ17zipR protein. (**A**) Mechanical strength, (**B**) Young’s modulus, (**C**) conventional yield strength.

The use of the recombinant EJ17zipR protein in the GelMa10% hydrogel and dECM bioink leads to significant changes in the mechanical parameters of the printed constructs (Figure 4). The mechanical properties of the construct should be similar to those of the tissue/organ repaired during regeneration [44,51,52,55,56,57,58,59,60]. It was observed that an increase in protein concentration in the biomaterial leads to an increase in the mechanical strength of the structure compared to the structure made of the reference material. Furthermore, material containing the EJ17zipR protein at a concentration of 1 mg/mL of 10% GelMa was not disrupted by the applied force. Such a bioink containing the EJ17zipR protein showed significant elasticity. Furthermore, the use of the EJ17zipR protein at a concentration as low as 0.1 mg/mL of bioink A led to an increase in the mechanical strength of the printed structure compared to the reference sample. The use of the recombinant EJ17zipR protein in dECM bioink A did not cause significant changes in the Young’s modulus of the printed structure compared to the reference sample.

**Figure 5:**
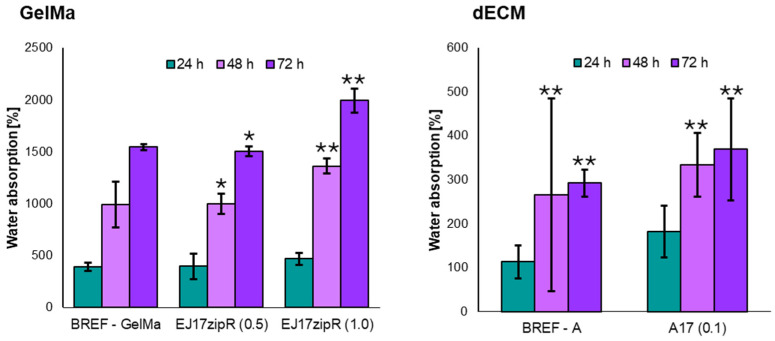
The degree of water absorption of materials based on GelMa and dECM containing the EJ17zipR recombinant protein (* *p* < 0.05, ** *p* < 0.0001).

The obtained results indicate that the use of a concentration of 0.1 mg of EJ17zipR in 1 mL of 10% GelMa has no effect on the degree of water absorption, while for a concentration of 1 mg/mL, an increase in the degree of water absorption was observed compared to the reference biomaterial. The addition of the EJ17zipR recombinant protein, regardless of the concentration in the tested range, resulted in an increase in the degree of water absorption compared to the reference bioink A. The results of the water absorption tests are presented in Figure 5. An increase in water absorption was observed at each measurement point. However, after 48 h of experimentation, the values obtained for the samples with added protein show a statistically significant increase. It was shown that the material containing (1.0) EJ17zipR recombinant protein showed almost 1.5 times higher absorption compared to the control material (GelMa) (*p* = 0.0004). The observed effect depends on the protein concentration used in the biomaterial. The material with a higher protein concentration showed an almost 30% increase in absorption properties compared to the material with a lower protein concentration (*p* = 0.0006). At the next time point (72 h), the significant advantage of the higher protein biomaterial was maintained. Compared to the control material, a difference of more than 20% was observed (*p* < 0.0001). Comparative analysis of the protein-enriched materials also showed an increase in water absorption of more than 20% for the material with the higher protein concentration (*p* < 0.0001). The bioink analysis confirmed the results previously obtained for GelMa with protein addition, and from the first measurement point, i.e., 24 h, a significant increase in absorption properties was already shown in the bioink with the addition of the tested recombinant protein. The obtained results suggest that the tested biomaterial could be used in the creation of three-dimensional structures of printed organs. The ability of the construct to absorb water is important in the case of, e.g., bone scaffolds, because it reflects the efficiency of absorption of body fluids and the transport of nutrients to cells [61,62,63]. Therefore, the use of the EJ17zipR protein could enhance the printing of structures of significant size while having a positive effect on the exchange of body fluids with the environment.

There are many additives that may improve the performance properties of bioprinting bioinks. The addition of the recombinant EJ17zipR protein is important for the rheological and mechanical properties and the printability of modern biomaterials. Moreover, it has a slight effect on water absorption.

**Figure 6:**
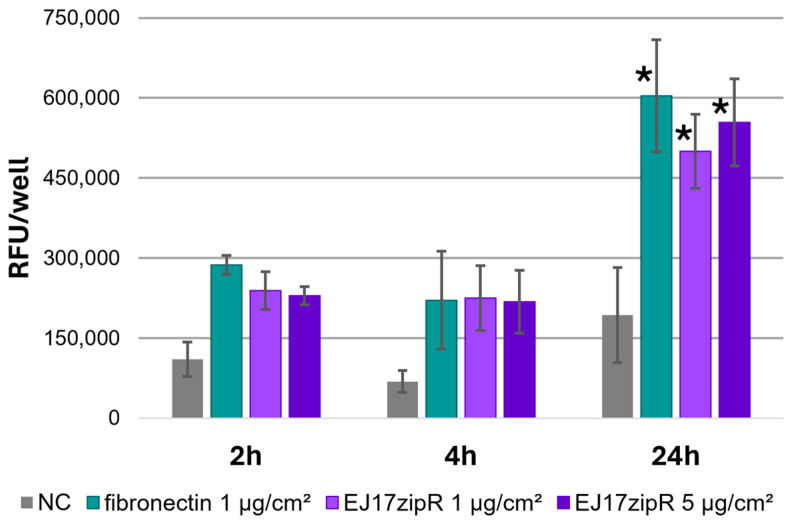
L929 cell adhesion test in the alamarBlue assay in plates coated with the EJ17zipR protein compared to fibronectin. The mean results of the three experiments are shown in the graph (* *p* < 0.05).

The tested EJ17zipR protein shows positive biological effects in the culture of the reference cell line L929. Adhesion assays showed an increase in the fluorescence level during incubation (Figure 6), confirming the adhesion of cells to the plastic plates used both in the positive control and in the wells coated with the tested protein. The effect of the concentration of the tested protein on the adhesion of the cells tested is clearly visible: with increasing protein concentration, the number of adherent cells increases after 24 h. However, it is worth noting that, despite the clear increase in the values obtained from as early as 2 h of the experiment, a statistically significant increase is observed after a day of the experiment. After 24 h, a more than 2.5-fold increase in adhesion was observed for 1 ug/cm^2^ vs. NC (*p* = 0.0024). However, as the concentration of protein used increased, the difference in adhesion was almost 3-fold (*p* = 0.0005).

**Figure 7:**
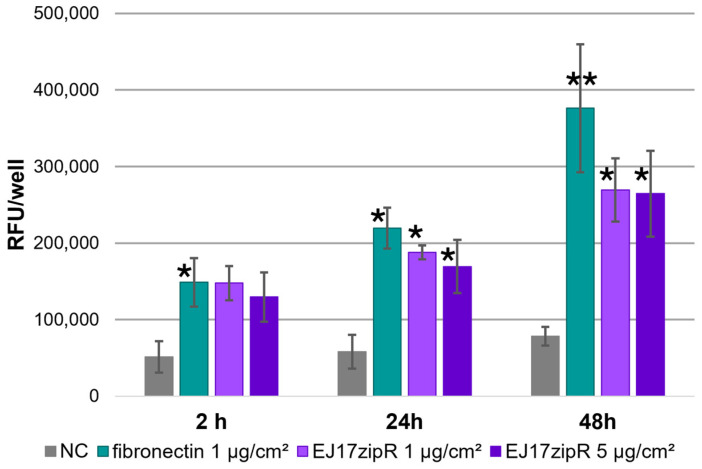
The proliferation assay with the L929 cell line with alamarBlue dying in plates coated with EJ17zipR and with fibronectin as a positive control. The graph shows the mean value of the results obtained in the three experiments (* *p* < 0.05, ** *p* < 0.0001).

In addition, both the positive control and the wells coated with the test protein showed active cell proliferation (the results of the experiment are shown in the graph in Figure 7). This is particularly evident after 24 h of the experiment, when an increase of more than 300% in the parameter tested was observed for the 1 μg/cm^2^ material (*p* = 0.0177) and an almost equal increase for the higher protein concentration (*p* = 0.0394). On the next day of the experiment, the differences were even greater, and for both tested protein concentrations they were more than three times higher than for the control sample (1 μg—*p* = 0.0010; 5 μg—*p* = 0.0013). The increase in fluorescence at 48 h compared to 2 and 24 h, taking into account the division time of the L929 cell line (about 24 h), confirms that the EJ17zipR protein does not interfere with the cell proliferation rate of the cell line used in this study. The slightly lower signals obtained for the test protein samples are probably due to lower cell adhesion to the EJ17zipR protein than to the fibronectin control.

**Figure 8:**
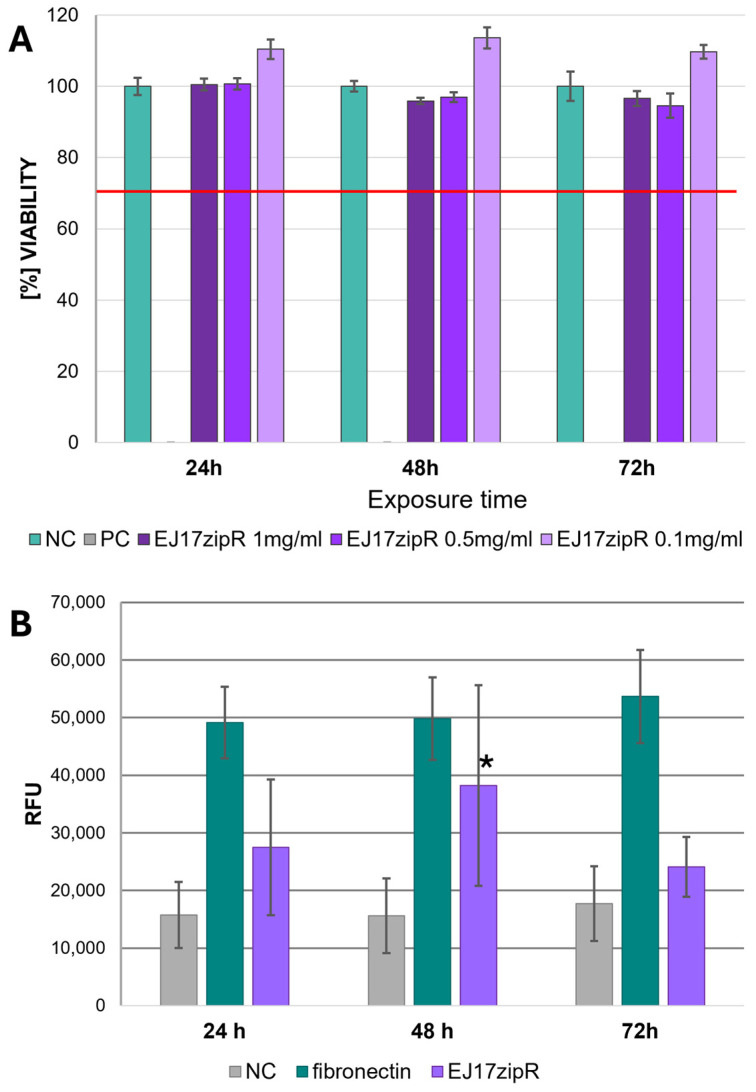
The cytotoxic effect of EJ17zipR protein as a solution on L929 cells—MTT assay (**A**)—and the cytotoxicity testing of EJ17zipR protein as a well coating on the L929 cell line compared to fibronectin (positive control). Green fluorescence measurement in calcein indicates living cells (**B**). (* *p* < 0.05).

In the cytotoxicity assays, it was concluded from the results that the EJ17zipR protein solution in the tested concentration range (0.1–1 mg/mL) was not cytotoxic to L929 fibroblasts in the MTT assay at 24, 48, and 72 h of exposure (Figure 8A). All samples tested in the MTT assay showed results above 70% viability (red line on the graph), indicating that the tested concentrations of EJ17zipR meet the acceptance threshold according to the medical device standard (a result > 70% indicates the absence of cytotoxic effect of the test sample against the cell line used in the assay). The increase in green fluorescence during incubation (Figure 8B) in calcein staining suggests that the EJ17zipR protein used to coat the culture plastic is not cytotoxic to cells of the L929 lineage. Statistical analysis of the results obtained after 48 h of incubation showed significantly higher values for the protein-enriched bioink. The difference between the control and the bioink with EJ17zipR protein added was almost 20% (*p* = 0.0329) although a significantly lower fluorescence signal was observed after 72 h of culture compared to the commonly used fibronectin.

This effect may be explained by the reduced number of cells adhering to the surface (microscopic observations), resulting in a reduced level of fluorescence derived from live cells. The results obtained in the experiments with the L929 cell line confirm previous observations that fibronectin-derived RGD sequences promote fibroblast adhesion and proliferation [31,32]. In the case of the EJ17zipR protein, two domains with a seven-repeat RGD sequence in the recombinant protein structure are responsible for the positive effect on the cell growth of the tested line. However, the level of cell growth observed after 48 h was lower than that in cells growing on the reference protein coating. This may be due to the spatial structure of the EJ17zipR protein and possible poor exposure of the central domain of the RGD sequence. In this scenario, only the C-terminal part of the protein with an exposed 7× RGD domain would be accessible to adherent cells. This result requires further studies on the spatial structure of EJ17zipR and its role in creating a cell microenvironment.

**Figure 9:**
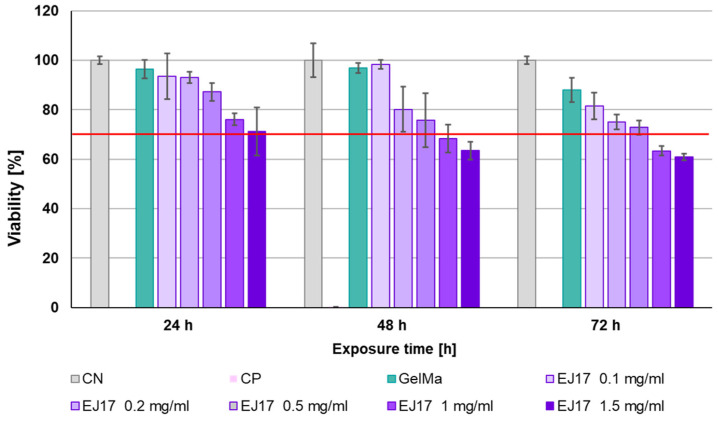
The viability of the L929 cell line in the presence of 10% GelMa supplemented with EJ17zipR protein—MTT indirect assay. The red line is an acceptance threshold of >70% viable cells after exposure to potentially cytotoxic material.

Since the EJ17zipR protein in the range of 0.1 to 1 mg/mL was not cytotoxic against the reference cell line L929 in direct contact, it was investigated whether a comparable effect could be obtained in the biomaterial. For this purpose, a colorimetric MTT assay was again performed, but an indirect method was used by adding recombinant protein to extracts of 10% GelMa at a concentration of 0.1 to 1.5 mg/mL of biomaterial. Lack of cytotoxicity was observed for all biomaterials tested after 24 h of exposure. During the second day of incubation, cell viability began to gradually decrease, with a lack of cytotoxicity still observed at 0.1 mg/mL–0.5 mg/mL. For GelMa with 1 or 1.5 mg/mL EJ17zipR, cell viability decreased below the reference standard (70%) on the second day of incubation. For biomaterials supplemented with 0.1 mg/mL–0.5 mg/mL EJ17zipR, the percentage of viability was still above 70% of the norm after 3 days of incubation, which demonstrates the lack of cytotoxicity of these materials (Figure 9). It can be concluded that an EJ17zipR protein content of less than ≤0.5 mg/mL in the test biomaterials is safe for the reference cell line. To sum up, the EJ17zipR protein is not only a valuable additive to bioink but also safe in terms of cell viability.

## 4. Conclusions

The design of the recombinant structural protein described in this paper was inspired by elastin, silk, and fibronectin sequences. The EJ17zipR protein was obtained in a prokaryotic expression system with high yield and efficiently purified in the original multistep process. Its amino acid sequence and high capacity to form oligomers were confirmed. The recombinant protein obtained demonstrates the physicochemical properties expected at the design level: we observed an improvement in high-resolution printing conditions. Clearly, the recombinant protein performed better than the reference material without the addition of EJ17zipR, producing an exceptional elasticity of final printouts. Moreover, the EJ17zipR protein supplement positively changed the rheological and mechanical properties of the tested biomaterials. In the study of the biological properties, clear results were obtained indicating a low level of cytotoxicity of the studied protein against the reference fibroblast cell line L929. Furthermore, a positive effect of the presence of the EJ17zipR protein on the adhesion and proliferation of the said cells was demonstrated. In conclusion, the results of biological studies indicate that the addition of the investigated protein to bioinks creates a favorable microenvironment for cell adhesion, growth, and migration in printed organoids and tissues. Due to its structural characteristics, EJ17zipR appears to be a promising biotechnological component of bioinks designed to print flexible and highly elastic spatially complex structures for regenerative medicine by positively influencing both shape stability and the internal transport of body fluids and nutrients.

## 5. Patents

Patent application resulting from the work reported in this manuscript: PCT/PL2024/050017.

## Data Availability

Data are contained within the article and Supplementary Materials.

## Supplementary Materials

The following supporting information can be downloaded at: https://www.mdpi.com/article/10.3390/jfb15060141/s1, Figure S1: Map of the expression vector EJ17zipR_pET-11a encoding the EJ17zipR hybrid protein gene sequence; Figure S2: Amino acid sequence of the recombinant hybrid protein EJ17zipR; Figure S3: EJ17zipR protein mass spectrometry analysis.
