# Supplementary material for: Elasticity Modification of Biomaterials Used in 3D Printing with an Elastin–Silk-like Recombinant Protein
Source: J Funct Biomater. 2024 May 24;15(6):141. doi: 10.3390/jfb15060141 (PMC11204424; doi:10.3390/jfb15060141)
Supplement: Supplementary file 1 [file jfb-15-00141-s001.zip › jfb-2962424-supplementary.pdf]

Supplementary materials

Figure S1: Map of the expression vector EJ17zipR_pET-11a encoding the EJ17zipR hybrid protein gene sequence.

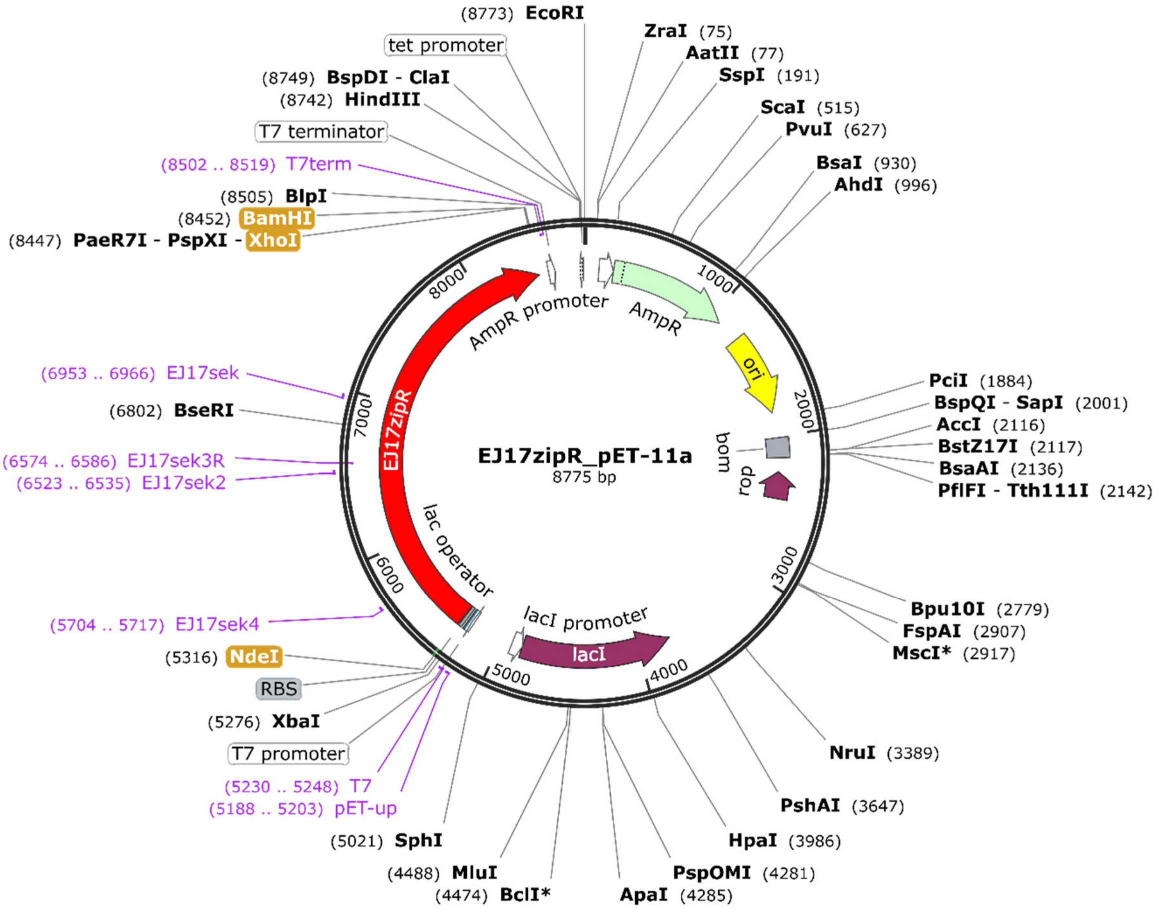

Figure S2: Amino acid sequence of the recombinant hybrid protein EJ17zipR

MVGGGGGKENQIAIRASFLEKENSALRQEVADLRKELGKCKNILAKYEAGGGGG

VPAGGVPAAGVPAAGVPAAGVPAFGVPAFGVPAGGVPAAGVPAAGVPAAGVPA

GGVPAAGVPAAGVPAAGVPAFGVPAFGVPAGGVPAAGVPAAGVPAAGVPAGGV

PAAGVPAAGVPAAGVPAFGVPAFGVPAGGVPAAGVPAAGVPAAGVPAGGVPAA

GVPAAGVPAAGVPAFGVPAFGVPAGGVPAAGVPAAGVPAAGVPAGGVPAAGVP

AAGVPAAGVPAFGVPAFGVPAGGVPAAGVPAAGVPAAGVPAGGVPAAGVPAAG

VPAAGVPAFGVPAFGVPAGGVPAAGVPAAGVPAAGVPAGGVPAAGVPAAGVPA

AGVPAFGVPAFGVPAGGVPAAGVPAAGVPAAGGAGAGSGAGAGSGAGAGSGA

GAGSGAGAGSGAGAGSGAGAGSGAGAGSGAGAGSGAGAGSAVTGRGDSPASSA

VTGRGDSPASSAVTGRGDSPASSAVTGRGDSPASSAVTGRGDSPASSAVTGRGDSPA

SSAVTGRGDSPASSVPAGGVPAAGVPAAGVPAAGVPAFGVPAFGVPAGGVPAAG

VPAAGVPAAGVPAGGVPAAGVPAAGVPAAGVPAFGVPAFGVPAGGVPAAGVPA

AGVPAAGVPAGGVPAAGVPAAGVPAAGVPAFGVPAFGVPAGGVPAAGVPAAGV

PAAGVPAGGVPAAGVPAAGVPAAGVPAFGVPAFGVPAGGVPAAGVPAAGVPAA

GVPAGGVPAAGVPAAGVPAAGVPAFGVPAFGVPAGGVPAAGVPAAGVPAAGVP

AGGVPAAGVPAAGVPAAGVPAFGVPAFGVPAGGVPAAGVPAAGVPAAGVPAGG

VPAAGVPAAGVPAAGVPAFGVPAFGVPAGGVPAAGVPAAGVPAAGGAGAGSGA

GAGSGAGAGSGAGAGSGAGAGSGAGAGSGAGAGSGAGAGSGAGAGSGAGAGS

AVTGRGDSPASSAVTGRGDSPASSAVTGRGDSPASSAVTGRGDSPASSAVTGRGDSP

ASSAVTGRGDSPASSAVTGRGDSPASS

Figure S3: EJ17zipR Protein Spectrometry Mass analysis

Mass spectrometry experiments were performed at the Mass Spectrometry Laboratory at the Institute of Biochemistry and Biophysics PAS.

The protein EJ17zipR-R were resuspended in 100ul 0.1% TFA in H_2O before mass measurement. Next samples were analyzed using LC-MS system Waters Acquity, column Acquity UPLC Protein BEH C4, 300A, 1.7um, 1mmX50mm nr cat 186005589 coupled to SYNAPT-G2. Due to problem with EJ17zipR-R mass measurement, the protein was first reduced by 1 hour incubation with 20 mM tris(2-carboxyethyl)phosphine (TCEP) and analyzed with LC-MS. In the second approach the protein was resuspended in strong detergent n-Dodecyl-β-D-maltoside (THERMO-89902). None of this methods gave positive results.

Protein identification

The dry submitted samples were resuspended in 100ul 0.1% TFA in H2O. Next, the cysteines were reduced by 1 hour incubation with 20 mM tris(2-carboxyethyl)phosphine (TCEP) at 37oC followed by 10 min incubation at a room temperature with 50 mM methyl methanethiosulfonate (MMTS). Digestion was performed 3h on immobilized pepsin (Thermo Scientific) at RT. After digestion, peptides were acidified with 0.1% formic acid.

Samples were analysed using LC-MS system composed of Evosep One (Evosep Biosystems, Odense, Denmark) coupled to a Orbitrap Exploris 480 mass spectrometer (Thermo Fisher Scientific, Bremen, Germany) via Flex nanoESI ion source (Thermo Fisher Scientific, Bremen, Germany). Samples were loaded onto disposable Evotips C18 trap columns (Evosep Biosystems, Odense, Denmark) according to the

manufacturer protocol with minor modifications. Briefly, Evotips were activated with 25 μl of 80% solution solvent B and 20% solvent A (solvent B: 0.1% formic acid in acetonitrile) by 1 min centrifugation at 600 g followed by 2 min incubation in 2-propanol. After equilibration with 25 μl of solvent A, 20 μl of each sample solution was loaded onto the solid phase. Bound peptides were washed with 50 μl and covered with 200 μl of solvent A. Chromatography was carried out at a flow rate 250 nl/min using the 88 min (15 samples per day) preformed gradient on EV1106 analytical column (Dr Maisch C18 AQ, 1.9 μm beads, 150 μm ID, 15 cm long, Evosep Biosystems, Odense, Denmark). Data was acquired in positive mode with a data-dependent method using the following parameters. MS1 resolution was set at 60 000 with a normalized AGC target 300%, Auto maximum inject time and a scan range of 300 to 1600 m/z. For MS2, resolution was set at 15 000 with a Standard normalized AGC target, Auto maximum inject time and top 40 precursors within an isolation window of 1.6 m/z considered for MS/MS analysis. Dynamic exclusion was set at 20 s with allowed mass tolerance of ±10 ppm and the precursor intensity threshold at 5e3. Precursor were fragmented in HCD mode with normalized collision energy of 30%. Spray voltage was set to 2.1 kV, funnel RF level at 40, and heated capillary temperature at 275 °C.

Raw data were pre-processed with the Mascot Distiller software (v. 2.4.2.0; Matrix Science), then obtained peptide masses and fragmentation spectra were matched to the E.coli database (4403 sequences; 1354446 residues), cRAP (115 sequences; 38188 residues) and User database, when study modifying sequences was added, using the

Mascot search engine (Mascot Daemon v. 2.4.0, Mascot Server v. 2.4.1, and Matrix Science). The following parameters were adopted for database searches: enzyme specificity was set to None, peptide mass tolerance to 5 ppm and fragment mass tolerance to 0.01 Da. The protein mass was left as unrestricted, and mass values as monoisotopic with two missed cleavages being allowed. Methylation of cysteine was set as fixed and oxidation of methionine was set as a variable modification. Protein identification was performed using the Mascot search engine, with the probability-based algorithm. The Decoy Mascot functionality was used for keeping FDR for peptide identifications below 1%.
